# The Dual Role of AQP4 in Cytotoxic and Vasogenic Edema Following Spinal Cord Contusion and Its Possible Association With Energy Metabolism via COX5A

**DOI:** 10.3389/fnins.2019.00584

**Published:** 2019-06-14

**Authors:** Yuan Huang, Sheng-nan Li, Xiu-ya Zhou, Li-xin Zhang, Gang-xian Chen, Ting-hua Wang, Qing-jie Xia, Nan Liang, Xiao Zhang

**Affiliations:** ^1^Clinical Laboratory Medicine, Chengdu Medical College, Chengdu, China; ^2^Center for Experimental Technology of Preclinical Medicine, Chengdu Medical College, Chengdu, China; ^3^Yunnan Forestry Institute, Kunming, China; ^4^Institute of Neuroscience, Kunming Medical University, Kunming, China; ^5^Institute of Neurological Diseases, Translational Neuroscience Center, West China Hospital, Sichuan University, Chengdu, China

**Keywords:** spinal cord injury, vasogenic edema, cytotoxic edema, AQP4, COX5A

## Abstract

Spinal cord edema, mainly including vasogenic and cytotoxic edema, influences neurological outcome after spinal cord contusion (SCC). Aquaporin 4 (AQP4) is the most ubiquitous water channel in the central nervous system (CNS), which is a rate-limiting factor in vasogenic edema expressing in brain injury, and it contributes to the formation of cytotoxic edema locating in astrocytes. However, little is known about the regulatory mechanism of AQP4 within vasogenic and cytotoxic edema in SCC, and whether the regulation mechanism of AQP4 is related to Cytochrome coxidase (COX5A) affecting energy metabolism. Therefore, the SCC model is established by Allen’s method, and the degree of edema and neuronal area is measured. The motor function of rats is evaluated by the Basso, Beattie, and Bresnahan (BBB) scoring system. Meanwhile, AQP4 and COX5A are detected by real-time quantitative PCR (qRT-PCR) and western blot (WB). The localization of targeted protein is exhibited by immunohistochemical staining (IHC) and immunofluorescence (IF). Additionally, the methodology of AQP4 lentivirus-mediated RNA interference (AQP4-RNAi) is used to reveal the effect on edema of SCC and the regulating molecular mechanism. Firstly, we observe that the tissue water content increases after SCC and decreases after the peak value of tissue water content at 3 days (*P* < 0.05) with abundant expression of AQP4 protein locating around vascular endothelial cells (VECs), which suggests that the increasing AQP4 promotes water reabsorption and improves vasogenic edema in the early stage of SCC. However, the neuronal area is larger than in the sham group in the 7 days (*P* < 0.05) with the total water content of spinal cord decrease. Meanwhile, AQP4 migrates from VECs to neuronal cytomembrane, which indicates that AQP4 plays a crucial role in aggravating the formation and development of cytotoxic edema in the middle stages of SCC. Secondly, AQP4-RNAi is used to elucidate the mechanism of AQP4 to edema of SCC. The neuronal area shrinks and the area of cytotoxic edema reduces after AQP4 downregulation. The BBB scores are significantly higher than in the vector group after AQP4-RNAi at 5, 7, and 14 (*P* < 0.05). There is a relationship between AQP4 and COX5A shown by bioinformatics analysis. After AQP4 inhibition, the expression of COX5A is significantly upregulated in the swelling astrocytes. Therefore, the inhibition of AQP4 expression reduces cytotoxic edema in SCC and improves motor function, which may be associated with upregulation of COX5A via affecting energy metabolism. Moreover, it is not clear how the inhibition of AQP4 directly causes the upregulation of COX5A.

## Introduction

Spinal cord injury (SCI) is currently an irreversible pathological condition that is characterized by devastating loss of functions in motor and sensory abilities of victims for life (Trgovcevic et al., 2014; Ahuja et al., 2017). The initial symptom is usually a series of secondary injuries characteristic of inflammation, apoptosis and edema in the cell. These secondary injuries result in consequential motor and sensory dysfunctions after SCI (Fujii et al., 1993; Flanders et al., 1999; McDonald and Sadowsky, 2002; Liu and Xu, 2012; Zhang et al., 2015). Spinal cord edema is the major initiating factor of SCI, which includes vasogenic edema and cytotoxic edema (Demediuk et al., 1990; Finnie et al., 2014). Recent studies have shown a mixed cytotoxic and vasogenic edema occurring in Subarachnoid Hemorrhage and photothrombotic model (Weimer et al., 2017; Uzdensky, 2018), but it is not clear whether there is any relationship in the occurrence of the two edema after SCI. Vasogenic edema is caused by excess fluid flowing to the extracellular space of spinal cord parenchyma due to the damage of tight junctions between vascular endothelial cells (VECs), blood-spinal cord barrier, and capillary walls in the models of SCI, as well as in cerebropathy and brain edema in rats (Beggs and Waggener, 1975; Griffiths, 1975; Okutan et al., 2008; Kim and Jung, 2011). Meanwhile, cytotoxic edema is caused by excess water influx to the cytoplasm of neurons and/or astrocytes leading to cell swelling (Rungta et al., 2015). Astrocytes are the key participants in spinal cord cytotoxic edema by virtue of their relationship with the spinal cord vasculature, functioning with their water transport proteins (Sofroniew and Vinters, 2010; Kitchen et al., 2015a). One of the consequences of spinal cord contusion (SCC) has been found to induce the expression of various genes relating to edema and inflammation, including Aquaporin 4 (AQP4) and cytokines (Schwab et al., 1998; Yuan et al., 2000; Schubert et al., 2008), and evidence shows that cellular water entry during cytotoxic edema is mediated by AQP4 channels in astroglial cells in traumatic brain injury (TBI) (Benfenati et al., 2011; Thrane et al., 2011; Hsu et al., 2015; Sturdivant et al., 2016).

AQP4 is the abundant aquaporin in the central nervous system (CNS) and is located in astrocytes and VECs as reported in previous studies (Nagelhus et al., 1998; Lu and Sun, 2003; Oshio et al., 2004; Mou et al., 2009). The deficiency of AQP4 in knock-out mice indicates slower water movement inside and outside of brains, resulting in opposite consequences for vasogenic and cytotoxic brain edema. Perivascular expression of AQP4 is considered as a rate-limiting factor in vasogenic edema formation. Nevertheless, when AQP4 surface expression increases in the astrocytic, it contributes to the change of membranal permeability for water molecules, which is subsequently conducive to the formation of cytotoxic edema (Hsu et al., 2015; Qiu et al., 2015; Mootaz et al., 2017b). The role of AQP4 in different types of SCC edema needs to be verified. Furthermore, it has been reported that AQP4-mediated water transport relies on the normal function of the sodium potassium pump, which is associated with cellular energy metabolism (O’Donnell et al., 2004; Lv et al., 2013). Interestingly, it is demonstrated that cytotoxic edema is probably associated with energy metabolism of the cells in CNS (Von Holst and Li, 2013; Von Holst and Li, 2015). Therefore, we aim to explore whether the role of AQP4 in cytotoxic edema is associated with energy metabolism and its possible mechanism.

The cytochrome c oxidase (COX5A) is a critical enzyme complex composing the respiratory electron transport chain, which is located in the mitochondria and participates in the metabolism process of cells. The participation of COX5A is essential for the production of adenosine triphosphate (ATP; Tsukihara et al., 1995; Carr and Winge, 2003; Alonso et al., 2003; Pecina et al., 2004). It is suggested that the expression of COX5A may be involved in cytotoxic edema by influencing the respiration and energy conversion of cells. So, whether the disorder of COX5A determines the formation of cytotoxic edema of SCI needs to be answered.

To our knowledge, it is difficult to directly knock down targeted genes *in vivo* with high selectivity and specificity. As a post-transcriptional gene silencing mechanism, RNA interference (RNAi) can be used in certain biological systems to interfere with the expression of endogenous genes (Nellen and Lichtenstein, 1993; Escors and Breckpot, 2010; High et al., 2014), which demonstrates great promise for the study of signal transduction research, human gene function, and gene therapy (Fire et al., 1998; Del Vecchio et al., 2019). Meanwhile, the lentivirus can stably integrate itself into the host’s genome and its remarkable packaging capacity also renders it available to gene transfer tools (Morris, 2006; Pluta and Kacprzak, 2009). The lentiviral vector may be a suitable delivery vehicle for RNAi technology, promoting the development of scientific research.

We hereby propose that AQP4 influences the formation of cytotoxic edema associated with the expression of COX5A. The first aim of this study is to observe whether vasogenic edema and cytotoxic edema occur simultaneously or successively in spinal cord tissues after SCC. The expression and localization of AQP4 and COX5A after SCC, respectively, are then detected by real-time quantitative PCR (qRT-PCR), Western blot (WB), immunohistochemistry (IHC) and immunofluorescence (IF). The second aim is to explore the role of AQP4 and whether AQP4 interference mediated by lentivirus affects edema following SCC. The extent of spinal cord edema after SCC is evaluated by wet/dry weight and the areas of neurons. The third aim is to verify a possible correlation in the interplay of AQP4 and COX5A, whether AQP4 interference mediated by lentivirus upregulates the expression of COX5A and thus impacts the cellular metabolism and ameliorates cytotoxic edema after SCC.

**Table 1:** Animal groups and lab investigation.

Groups	Methods				Total
	Water quantity	Morphology	qRT-PCR	WB	
Sham	6 × 3	3	0	8	29
SCC	6 × 3	3 × 8	0	8 × 4	74
Vector	0	3 × 8	8 × 3	0	48
AQP4-RNAi-LV	0	3 × 8	8 × 3	0	48
Total	36	75	48	40	199

## Materials and Methods

### Animal Grouping and SCC Animal Models

#### Animal Grouping

The SCC study was conducted in accordance with the National Institutes of Health guidelines for the treatment of animals and all experiments were approved by the Institutional Animal Care and Use of Laboratory Animals of Sichuan University, Chengdu, China. Adult female Sprague–Dawley rats (*n* = 199) weighing 225–250 g were randomly divided into four groups of varying numbers for the following procedures: (1) sham group (laminectomized only); (2) SCC groups (T9 SCC only); (3) vector group (T9 SCC and vehicle vector injection); (4) AQP4-RNAi-LV group (T9 SCC and AQP4-RNAi-LV injection). Rats were subjected to SCC induced by Infinite Horizon Device (Chengdu Taimeng Company). Animals were allowed to survive 6 h, 12 h, 1 day, 3 days, 5 days, 7 days, 14 days, and 28 days. The specific animal groups and lab investigation are shown in Table 1.

#### Animal Treatment

In order to study the mechanism of SCC *in vivo*, animal models of SCC were prepared by the modified Allen’s test. Rats were anesthetized by intraperitoneal injection of 3.6% chloral hydrate (1 ml/100 g) and fixed in a supine position, then underwent a partial laminectomy of the 10th thoracic vertebraes (shown in Supplementary Figure S1). Spinal cord lesions were inflicted by an Infinite Horizon Device through 0.15 N (10 g × 3 cm × 9.8 N/kg) force (shown in Supplementary Figure S2). The sham group was performed with laminectomy without damage of the spinal cord. The surgical site was closed by suturing the muscle and fascia as well as the skin. Subsequently, rats were injected intraperitoneally with 2 ml of 0.9% sterile saline and placed on a heating pad to maintain body temperature until they were revived. Then, rats were transferred to a bacteria-free biologically clean room and provided with water and food. Rats were also injected intraperitoneally with 2 ml of 4.8% penicillin once a day in the initial 3 days after surgery. Bladders of all injured rats were manually emptied twice daily until normal function was recovered. All procedures were in compliance with the recommendations in the University Committee on Animal Use and Care. We used the minimum number of animals necessary for each experiment and ensured that the suffering of animals was minimized.

#### Neurobehavioral Assessment

The recovery of hind limb motor function was evaluated by the Basso, Beattie, and Bresnahan (BBB) scoring system (Basso et al., 1995). Rats (*n* = 6) were randomly selected from each group for evaluation at corresponding survival time points. The animals were allowed to move freely in a 1 m × 1 m field. The ipsilateral hind limb motor functions were evaluated with a 21-point scale. A score of 21 means complete mobility and a score of 0 means complete paralysis. All measurements were obtained using a double-blind method by five observers. Four of the observers acquire points, and the other one acts as a quality controller (removed one score in which the difference was too big) and data recorder. Every rat was assessed over 5 min and scores were acquired from three evaluators. The average score was recorded as the final result.

### Bioinformatical Analysis

The bioinformatical analysis was used to predict the relationship among AQP4 and other genes according to the information from the Internet^1^ for rat species.

**Table 2:** Sequence information of AQP4 siRNA.

F1 target sequences	*CCAAGTCCGTCTTCTACAT* *5′ CCAAGUCCGUCUUCUACAU dTdT 3′* *3′ dTdT GGUUCAGGCAGAAGAUGUA 5′*
F2 target sequences	*CAGGTGCACTTTACGAGTA* *5′ CAGGUGCACUUUACGAGUA dTdT 3′* *3′ dTdT GUCCACGUGAAAUGCUCAU 5*
F3 target sequences	*CAGCATGAATCCAGCTCGA* *5′ CAGCAUGAAUCCAGCUCGA dTdT 3′* *3′ dTdT GUCGUACUUAGGUCGAGCU 5′*

### Construction of AQP4 Lentiviral Expression Vectors

#### Preparation of AQP4 siRNA

Three targeted sequences of AQP4 siRNA were designed and synthesized by Ruibo Company Guangzhou, China. The sequence’s information is shown in Table 2. We examined AQP4 gene expression to find the siRNA with the highest interference efficiency in the 293T system *in vitro*.

#### Preparation of Recombinant Lentiviral Vector

To determine the mechanism of AQP4 activity, the AQP4 lentiviral vector was made. We examined the gene expression of AQP4 to find the exact siRNA with the most significant interference effect in the 293T system *in vitro*. Information on the siRNA fragment with the highest interference efficiency was provided by GeneCopoeia (GuangZhou, China) and applied to construct the AQP4-lentiviral expression vector. Meanwhile, the vector expressed a gene encoding a red fluorescent protein (RFP). Thereafter, AQP4 interference vector (5 μg) and viral packaging vectors (1 μl, GeneCopoeia, GuangZhou, China) were co-transfected into 293T cells to produce lentiviral particles (AQP4-RNAi-LV). The viral supernatant was harvested at 48 h post-transfection and filtered through a 0.45 μm cellulose acetate filter. Then, 5 ml cell supernatant containing lentivirus was centrifuged (3500× *g*, 25 min), and the precipitate was re-dissolved in 500 μl PBS. Finally, the lentivirus was frozen at −80°C. The negative lentiviral vector was also packaged and used as a negative control, which has no effect on any gene theoretically.

#### Injection of AQP4 Interference Lentivirus Into the Spinal Cord Tissue *in vivo*

Spinal cord contusion rats were randomly assigned to a vector group and an AQP4 interference lentiviral vector group (AQP4-RNAi-LV group). After being deeply anesthetized with 3.6% chloral hydrate (1 ml/100 g), the rats were fixed in a stereotaxic apparatus. The T9 segment of the spinal cord was exposed after laminectomy, and an equal volume of lentivirus or negative vector (4 μl per rat) was injected into two points on a coronal plane of the T9 segment of the spinal cord with a microsyringe (Hamilton) (shown in Supplementary Figure S4). The skin was sutured after surgery. Animals were given the optimum temperature and maintained in comfortable cages. Then, 48 h after the injection, animals received SCC, as describe as above.

#### Sample Preparation

At the scheduled time points of SCC, rats were transcardially perfused with ice-cold 0.9% saline. Then, spinal cord samples were harvested and the protein was extracted to confirm the lentivirus’s interference efficiency by Western blotting. Generally, tissue that expressed red fluoresce protein was deemed to be transfected successfully in the spinal cord by AQP4-RNAi-LV. It was noteworthy that the diffusion location of lentivirus was approximately 5 mm^2^. Therefore, 3 mm^2^ of spinal cord tissue surrounding the epicenter of injury was removed for Western blotting analysis.

#### Verifying Interference Efficiency of AQP4-RNAi-LV by Western Blotting Analysis

Spinal cord tissue transfected by AQP4-RNAi-LV was collected to extract protein, which was used to assess the protein expression of AQP4 following lentiviral injection. Protein samples were boiled for 10 min. The proteins fractionated on 15% SDS-PAGE gels and were transferred to polyvinylidene difluoride membranes (PVDF) and Millipore membranes for 120 min at 75 V by electrophoresis. The membranes were then blocked in 5% non-fat milk for 120 min at room temperature and incubated with primary antibody of AQP4 (rabbit, ab46182, Santa Cruz, 1:200) overnight (16–20 h) at 4°C. After incubation with the primary antibody, the membranes were washed in three changes of buffered saline TBST and incubated for 120 min with secondary antibody (HRP anti-rabbit IgG, ZSGB-BIO, diluted by 1:5000. Finally, membranes were rinsed in TBST 3 times and the immune complexes were visualized with enhanced chemiluminescence (ECL) and captured on Alpha Innotech (Bio-Rad). Band intensities were determined using densitometry (ImageJ), and AQP4 levels were normalized to beta-actin.

### Methods of Detection

#### Detection of Water Quantity in the Spinal Cord

To determine the extent of edema of the spinal cord, tissues were assessed in the sham, 1, 3, and 7 days groups after injury using the wet/dry weight method (Ni et al., 2015). Briefly, animals were administered with a lethal dose of pentobarbital and the spinal cord was removed rapidly. The spinal cord was cut into 10 mm segments to obtain the wet weight, and then the spinal cord tissues were oven dried at 100°C for 48 h to measure the dry weight. The water content of spinal cord (as a percentage) was calculated as [(wet weight–dry weight)/wet weight] × 100%.

#### Detection of the Area of Motor Neurons in Spinal Cord

The area of motor neurons in the anterior horn of the spinal cord was detected by ImageJ. In the sham, 1, 3, 7, and 14 days groups, the area of motor neurons was used to show the degree of cytotoxic edema after SCC. We used ImageJ software to draw the shape of neurons, which auto-calculated the area of shape drawing.

**Table 3:** Detailed information on the selection of primers for RT-PCR experiments.

Name	Primers sequence (sense)	Primers sequence (antisense)
β-actin	*GAAGATCAAGATCATTGCTCCT*	*TACTCCTGCTTGCTGATCCA*
AQP4	*GACATTTGTTTGCAATCAAT*	*AACCCAATATATCCAGTGGTT*
COX5A	*CGGTTAAATGATTTTGCTAGT*	*CACTTTGTCAAGGCCCAGTT*

#### Real-Time Quantitative Polymerase Chain Reaction

To detect the expression level of AQP4 and COX5A mRNA in the spinal cord, qRT-PCR was used. Total RNA was extracted from the spinal cord tissue of rats by Trizol reagent (Superfec TRI) according to the manufacturer’s protocol and reversely transcripted to cDNA with the RevertAid^TM^ First Strand cDNA Synthesis kit (TaKaRa Biotechnology, Dalian, China). qRT-PCR was then performed to determine the expression levels of target genes. The primer sequences are shown in Table 3. qRT-PCR was performed in a DNA thermal cycler (ABI 7300) according to the following standard protocol: one cycle of 95°C for 2 min, 40 cycles of 95°C for 15 s, annealing at 55°C for 20 s and extending at 60°C for 40 s. Relative expression quantities were calculated with normalization to β-actin values by using the 2^−ΔCt^ method.

#### Western Blotting

In order to investigate the possible changes in AQP4 and COX5A proteins in the spinal cord with different treatments, Western blot was used. At the scheduled time points, the rats were deeply anesthetized with an intraperitoneal injection of 3.6% chloral hydrate (1 ml/100 g) and were transcardially perfused with ice-cold 0.9% saline. Then, the spinal cords of the lesion site (0.5 cm) were harvested and homogenized ice-cold in 400 μl of RIPA buffer (Thermo Fisher Scientific) containing a cocktail of phosphatase and protease. Lysates were centrifuged at 12,000 × *g* for 30 min. The supernatant was extracted and stored at −80°C for later use. Protein concentration of each sample was assayed with BCA reagent (Sigma, St. Louis, MO, United States). A 20 μl aliquot of the samples was loaded and electrophoresed on 8 and 12% SDS polyacrylamide gel for 1.5 h at 120 V. The proteins were transferred from the gel to polyvinylidene difluoride membranes. Then the membranes were blocked by 5% nonfat dry milk for 120 min. Primary antibody of β-actin (Santa Cruz, sc-47778, 1:1000), anti-AQP4 (rabbit, Santa Cruz, sc-20812, 1:200) or anti-COX5A (mouse, abcam, ab180129, 1:100) was incubated overnight at 4°C. Afterward, the membranes were rinsed in phosphate-buffered saline (PBS) containing 0.05% Tween-20 three times. Then the membranes were incubated for 2 h with a HRP-conjugated goat anti-mouse IgG (1:5,000, ZSGB-BIO, SCCna,ZB-2301) or goat anti-rabbit IgG (1:5,000: ZSGB-BIO, SCCna, ZB-2308) for 2 h at room temperature. Finally, membranes were rinsed in the buffer three times and the immune complexes were visualized with ECL (Perkin Elmer) by Alpha Innotech (Bio-Rad). The densities of bands on immunoblots were measured by a Bio-Gel Imagining System equipped with Genius synaptic gene tool software for further quantitative analyses.

#### Immunohistochemistry

To determine the sites and effects of AQP4 and COX5A in morphology after SCI, immunohistochemistry was used. After anesthesia, the rats were perfused with 4% paraformaldehyde solution for 30 min. The injury sites of spinal cord both in the sham group and the SCC group were harvested. After being infiltrated in 4% paraformaldehyde solution for a postfix for 48 h, the spinal cord samples were embedded into one paraffin block, named as tissue array. The tissue array was sectioned into 5 μm slices. The original design of the tissue array was useful to detect the expression of AQP4 in different time points under identical conditions. Therefore, it was convenient to make a comparison of different groups. After the tissues were through hydration and deparaffinization, endogenous peroxidase activity was quenched with 0.3% (v/v) hydrogen peroxide for 15 min at room temperature. Non-specific adsorption was minimized by incubating the section in 2% (v/v) normal goat serum in PBS for 30min at 37°C. Then sections were incubated at 4°C overnight with polyclonal anti-AQP4 antibody (rabbit, abcam, ab9512, 1:100) or anti-COX5A (mouse, abcam, ab156445, 1:100). Sections were washed with PBS and incubated with biotinylated goat anti-rabbit IgG antibody (abcam, ab191866, 1:100) for 0.5 h at 37°C. Immunoreactivity was visualized as brown staining with a biotin-conjugated goat anti-rabbit IgG was and observed with a light microscope. Slices were pictured and assessed by densitometry as described earlier by using ImageJ software.

#### Immunofluorescence Analysis

Immunofluorescence was used to detect the location of AQP4 and COX5A expression in the spinal cord. Spinal cord tissues were obtained and placed in 20% sucrose solution in 0.1 M phosphate buffer (PB). After the specimens sank to the bottom of the bottle, they were placed on a freezing microtome (Leica CM1900, Wetzlar, Hesse, Germany) and cut into serial horizontal sections with 20 μm thickness. The primary antibodies anti-AQP4 (rabbit, sc-20812,Santa Cruz, 1:200) and anti-COX5A (mouse, abcam, ab180129, 1:100) were incubated with the sections at 4°C for 24 h. After washing, sections were incubated with fluorescence secondary antibody IgG (anti-rabbit 488: green or anti-mouse 594: red) for 4 h at 37°C. The control sections were incubated with PBS. After mounting, slides were observed by fluorescence microscopy. Negative control was performed without the primary antibody. Afterward, cell nuclei were visualized by DAPI-Fluoromount. For double labeling, anti-NEUN was chosen to adapt to species of anti-AQP4/anti-COX5A.

### Statistical Analysis

All data were presented as means ± standard error (Mean ± SEM). All value analysis was performed with SPSS 17.0 statistical software. One-way analysis of variance (ANOVA) was applied to multiple-group comparison. The significance of the difference between groups was calculated according to Fisher’s least significant difference *post hoc* test. Probability values (*P*) of less than 0.05 were considered to represent statistical significance.

## Results

### Evaluation of Motor Function Damage and Expression of AQP4 After SCC

#### Variations of Motor Function After SCC

**FIGURE 1:**
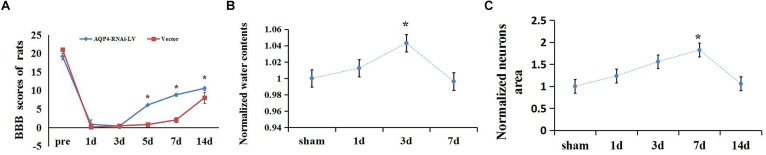
Extent of edema in spinal cord and changes of BBB scores after SCC. **(A)** The scores at 7, 14, and 28 days increased significantly compared to 3 days (*n* = 6, *P* < 0.05) in the SCC group. **(B)** Date presented as fold changes normalized to sham. The spinal cord tissue water content is recorded (70.17 ± 0.38)% in the sham group. At 1 day post-SCC, the percentage of water content increased to (71.04 ± 0.41)% (*n* = 6, *P* > 0.05) within the injury epicenter. A further increase was recorded at 3 days (73.19 ± 0.3)%, while on day 7 (69.90 ± 0.44)% (*n* = 6, *P* < 0.05) a decline appeared, compared with day 3. ^∗^denotes *P* < 0.05, compared with the sham group. **(C)** Date presented as fold changes normalized to sham. The area of motor neurons clearly increased in the anterior horn of the spinal cord at 3, 7, and 14 days after SCC, which showed the degree of cytotoxic edema. ^∗^*P* < 0.05 when compare to sham group (*n* = 6).

Basso, Beattie, and Bresnahan scale was recorded to evaluate the motor function in the hindlimbs of SCC and sham rats. In sham rats, the scores were 21, while the rats’ movement ability of hindlimbs disappeared (marked 0) in the early stage after SCC (shown in Supplementary Figure S3). It went on a gradual increase on BBB score, showing limited recovery as time passed. Moreover, the scores at 7, 14, and 28 days increased significantly compared to 3 days (*n* = 6, *P* < 0.05; Figure 1A). The original BBB scores after SCC shown in Supplementary Table S2.

#### Two Types of Edema Occur at Different Stages After SCC

To detect the edema extent of spinal cord after SCC, a wet/dry weight method was used. Water quantity of the spinal cord tissues in the sham group was recorded (70.17 ± 0.38)%, and it was lower than that of the SCC group at 3 days after injury (73.19 ± 0.19)% (Figure 1B, *P* < 0.05). This result showed significant variation in spinal cord edema after SCC. The original data of wet-dry weight after SCC shown in Supplementary Table S5.

To detect the extent of edema in the motor neurons after SCC, the areas of neurons were measured in the anterior horn of the spinal cord. The result showed that the areas of motor neurons increased constantly from 1 to 7 days after SCC. It increased to a peak value of 5.31 ± 2.25 (×100 μm^2^) at 7 days, which was significantly higher than that of the sham group (*P* < 0.05) (Figure 1C). This indicated that the cytotoxic edema in the spinal cord was aggravated obviously after SCC. The original data of neurons area shown in Supplementary Table S3.

#### Expression Increases and Translocation of AQP4 After SCC

**FIGURE 2:**
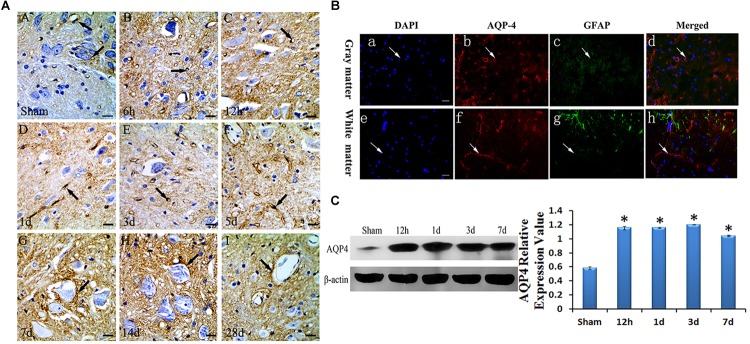
Expression and location of AQP4 in edema after SCC. Panel **(A)** shows the anterior horn of spinal cord gray matter (T10). AQP4 immunoreaction was in the gray matter of the spinal cord, which was revealed to be brown, we can see AQP4 increased apparently in SCC, and reached the peak at 3 days post-SCC, then has a transitory decrease, but at 7 days post-SCC, it picked up a lot, and was restored to preoperative level, at 28 days post-SCC. (**A**: sham group, showed the AQP4 within normal level. Panels **(B–I)** showed the AQP4 at 6 h, 12 h, 1 day, 3 days, 5 days, 7 days, 14 days and 28 days post-SCC). **(B)** Immunofluorescence was used to detect the distribution of AQP4 in the spinal cord at 3 days after SCC. Panels **(B-a–d)** show that DAPI, AQP4 and GFAP merge in the gray matter of the spinal cord. Panels **(B-e–h)** show that DAPI, AQP4 and GFAP merge in the white matter of spinal cord. Panels **(B-a,e)** show that the nucleus of cells was labeled by DAPI (blue) in the spinal cord. Panels **(B-b,f)** show AQP4 immunoreaction products (red). Panels **(B-c,g)** show that little of the GFAP immunolabeling (green) was detected in the somata and neuritis of astrocyte. AQP4 immunoreaction products (red) in the spinal cord were labeled in the VEC of gray matter. Panels **(B-d,h)** co-localization of DAPI (blue), AQP4 (red) and GFAP (green) are shown in the injured spinal cord, where AQP4 surrounds the VEC and in the astrocytic processes in injured spinal cords. Bar = 100 μm. **(C)** The bands show the immunostaining of AQP4 and beta-actin by Western blotting. The density of the bands measured by ImageJ. Data were normalized to beta-actin. The expression of AQP4 protein was significantly increased compared sham group at 12 h, 1 day, 3 days and 7 days after SCC. ^∗^*P* < 0.05 when compared to the sham group (*n* = 8).

To confirm the location of AQP4 in the spinal cord, immunohistochemistry and immunofluorescence were used. AQP4 immunoreaction positive products (red) were labeled in VECs as well as soma and axon of astrocytes, surrounded by VECs in the gray matter of the injured cord at 3 days after SCC (Figure 2B). The nucleus of the cells was labeled by DAPI (blue) in the gray matter of the spinal cord (Figure 2B-a). GFAP (green) showed the presence of astrocyte in the spinal cord (Figure 2B). In addition, AQP4 immunoreaction positive products (brown) were shown in Figure 2A. Consecutive monitoring about the localization of AQP4 was shown until 28 days after injury, and AQP4 was highly concentrated in the VECs and the glial cells in the gray of spinal cord. However, starting from 7 days it was seen obviously in the neuronal membranes and glial cells (Figure 2A). With the translocation of AQP4 from VECs to the membranes of neurons, the area of neurons in spinal cord gray matter was larger than it was before.

To determine the expressive quantity of AQP4 after SCC, Western blotting was used to detect it at sham, 12 h, 1 day, 3 days, and 7 days as shown in Figure 2C. Data was normalized by beta-actin. The optical density value of bands significantly increased (*P* < 0.05) at 12 h, 1 day, 3 days, and 7 days when compared to that of the sham group (Figure 2C).

### The Results of AQP4 Inhibition Mediated by Lentivirus in SCC

#### Identification of Oligonucleotide Sequences for Lentivirus-Mediated AQP4 Inhibition

**FIGURE 3:**
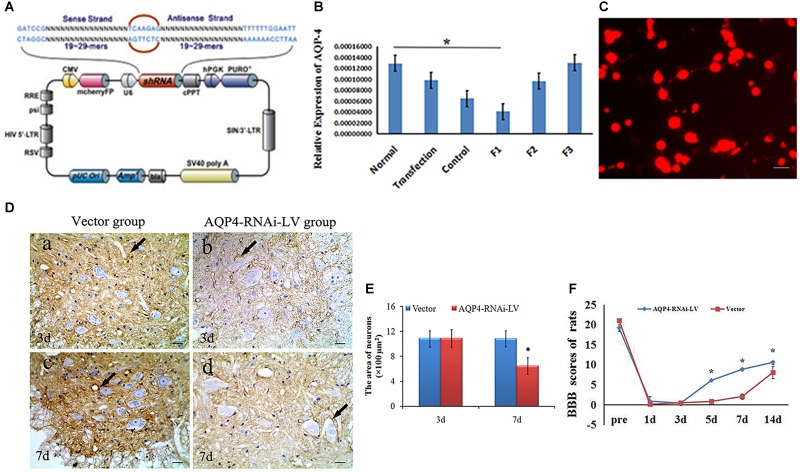
Preparation and identification of AQP4-RNAi-LV recombinants. **(A)** The schematic of the AQP4 siRNA sequence is shown. Enhanced red fluorescent protein (mcherryFP) as a reporter gene was inserted in the plasmid. The framework also contained the antibiotic ampicillin and pUC Ori promoters for vector expression. **(B)** qRT-PCR showed F1 was the most effective interference of AQP4. ^∗^*P* < 0.05 when compared to control group. **(C)** Fluorescent image of AQP4-RNAi-LV (red) transfected into 293T cells. **(D)** Immunohistochemistry shows the AQP4 immunoreactive products in the vector group and the AQP4-RNAi-LV group. Panel **(D-a)** shows AQP4 immunoreactive products in spinal cords of the vector group, and Panel **(D-b)** shows the AQP4 immunoreactive products in spinal cords of the AQP4-RNAi-LV group. It is clear that the degree of color in the vector group was stronger than the AQP4-RNAi-LV group, which suggests that the AQP4 interference affected the expression of AQP4 *in vivo*. Bar = 100 μm. **(E)** The area of neurons was measured by ImageJ, and the data showed that the area of neurons in the AQP4-RNAi-LV group was significantly decreased compared to the vector group at 7 days. ^∗^*P* < 0.05 when compared to vector group. **(F)** The BBB scores in the AQP4-RNAi-LV group were significantly higher than in the vector group at 5, 7, and 14 days (*n* = 6, *P* < 0.05). Values are expressed as mean ± SEM ^∗^*P* < 0.05 when compared to vector group.

In order to elucidate the mechanism of AQP4 in rats with SCC, we constructed the recombinant of AQP4. Three siRNA sequences for AQP4 (F1, F2, F3) were transfected into a 293T cell line to select the most effective siRNA segment by qRT-PCR (Figure 3A). Results showed that the expression of AQP4 in F1, F2 and F3 was 0.411, 0.767, and 1.035, respectively, suggesting that F1 was the most effective AQP4-siRNA sequence (Figure 3B). Immunofluorescent staining showed that the expression of AQP4 was labeled by RFP (red) in cells, which suggested that the AQP4-RNAi-LV had been successfully transfected into the host cells *in vitro* (Figure 3C).

After AQP4-RNAi-LV was injected into the spinal cord, the effectiveness of AQP4 interference was confirmed. Immunohistochemistry showed that the AQP4 immunoreactive positive products could be seen in the vector group and AQP4-RNAi-LV group. Furthermore, the deepness of color in the vector group was stronger than it was in the AQP4-RNAi-LV group (Figure 3D). Results showed that the expression of AQP4 could be interfered by injecting AQP4-RNAi-LV into spinal cord.

#### Lentivirus-Mediated Inhibition of AQP4 Ameliorates the Swelling of Neurons in SCC

The effect of AQP4 inhibition was detected by immunohistochemistry firstly, which was performed to assess the morphological changes in the spinal cord tissue following the injection of AQP4-RNAi. Compared to the vector group, the area of neurons was decreased (*P* < 0.05) in the AQP4-RNAi-LV group compared to the vector group at 7 days (Figure 3D,E). The results showed that inhibition of AQP4 mediated by lentivirus ameliorated the swelling of neurons in SCC.

#### Inhibition of AQP4 Facilitates Motor Function Recovery After SCC

Basso, Beattie, and Bresnahan test was used to evaluate the motor function recovery in hindlimbs following SCC. The BBB scores in the AQP4-RNAi-LV group were significantly higher than those in the vector group at 5, 7, and 14 days (*n* = 6, *P* < 0.05; Figure 3F). The result revealed that inhibition of AQP4 could accelerate functional recovery in the hindlimbs after SCC. The original BBB score after AQP4-RNAi shown in Supplementary Table S1.

### Correlation of AQP4 and COX5A in Cytotoxic Edema After SCC

#### AQP4 Correlates With COX5A by Bioinformatics Analysis

**FIGURE 4:**
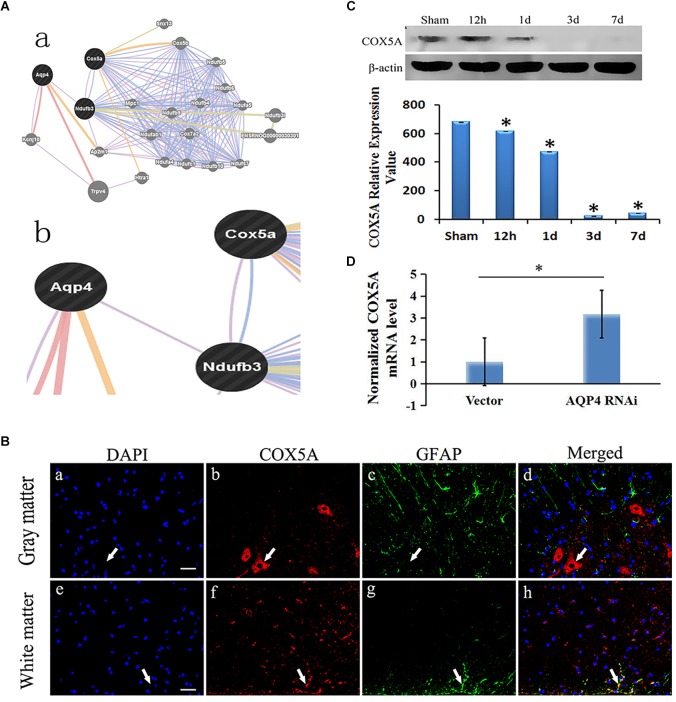
Correlation of AQP4 and COX5A in cytotoxic edema with SCC. **(A) (A-a)**, Detailed analysis shows the results of bioinformatical analysis. **(A-b)**: part of the bioinformatical analysis showed that AQP4 was co-expressed and co-localized with NADH dehydrogenase (ubiquinone) 1 beta subcomplex 3 (Ndufb3), COX5A and Ndufb3. **(B)** Immunofluorescence analysis demonstrated that COX5A (red) emerged in the anterior horn of spinal cord at 3 days after SCC. **(B-a–d)**: co-expression of GFAP and COX5A in the gray matter of the spinal cord. **(B-e–h)**: co-localization of GFAP and COX5A in white matter of spinal cord. **(B-a)**: the nuclei were labeled by DAPI (blue). **(B-b)**: COX5A immunoreaction (red). **(B-c)**, GFAP immunoreaction (green). **(B-d)**: merged (yellow) shows co-expression of DAPI and COX5A. **(B-e)**: the nuclei are in white matter by DAPI (blue). **(B-f)**, COX5A immune reactions (red). **(B-g)**, GFAP-positive neurons, green. **(B-h)**, Co-localization of COX5A and GFAP (yellow). Bar = 40 μm. **(C)** Western blotting shows the immunostaining of COX5A and beta-actin at sham, 12 h, 1 day, 3 days and 7 days. The density of bands was measured by ImageJ. Data were normalized to beta-actin. The expression of COX5A protein was significantly decreased compared to the sham group at 12 h, 1 day, 3 days, and 7 days after SCC. ^∗^*P* < 0.05 when compared to sham group (*n* = 8). **(D)** Following AQP4 lentivirus transduction *in vivo*, the expression of COX5A was detected by qRT-PCR. The level of COX5A was significantly increased in the AQP4-RNAi-LV group compared to the vector group. ^∗^*P* < 0.05 when compared to vector group (*n* = 8).

The correlation of AQP4 and COX5A could be revealed some way by bioinformation analysis, in which both were co-expressed via the introduction of Ndufb3(NADH ubiquinone oxidoreductase subunit B3) (Figure 4A).

#### COX5A Locates in the Anterior Horn of Spinal Cord After SCC

Using immunofluorescent analysis, we demonstrated that COX5A were observed in the anterior horn of the spinal cord at 3 days after SCC. However, COX5A was observed without obvious co-localization of astrocyte antigen (GFAP). Combined with morphology observation, the results indicated that COX5A was particularly enriched in the cytoplasm, soma and the proximal apical dendrite of neurons, especially in large motor neurons in the anterior horn of the spinal cord gray matter (Figure 4B).

#### The Expression of COX5A Decreases in the Spinal Cord After SCC

Using Western blotting, we observed different protein expression between AQP4 and COX5A in the gray matter of the spinal cord after SCC, with a decreased COX5A. It dropped to the lowest level at 3 days (23.59 ± 0.75, *P <* 0.05), a significant difference compared to the sham group, and a slight increase was recorded until 7 days (43.66 ± 0.72, *P* > 0.05) (Figure 4C). The original Western blot results of AQP4 and COX5A after SCC shown in Supplementary Material Table S6.

#### AQP4 Inhibition Upregulates the mRNA Expression of COX5A in SCC

Using qRT-PCR, we identified the relative expression of COX5A mRNA in the AQP4-RNAi-LV and vector groups. Results indicated that COX5A increased following AQP4-RNAi lentivirus transduction, with a significant difference at 7 days in SCC (*P <* 0.05, Figure 4D). The original qRT-PCR results shown in Supplementary Material Table S4.

#### COX5A Expression Upregulates and Neuronal Edema of SCC Improves After AQP4 Inhibition

To determine the localization of COX5A in motor neurons, IHC was performed following AQP4-RNAi lentivirus transduction. The results showed that the group of AQP4-RNAi-LV had more COX5A-positive products in motor neurons than the vector group at 3, 7, and 28 days after SCC.

**FIGURE 5:**
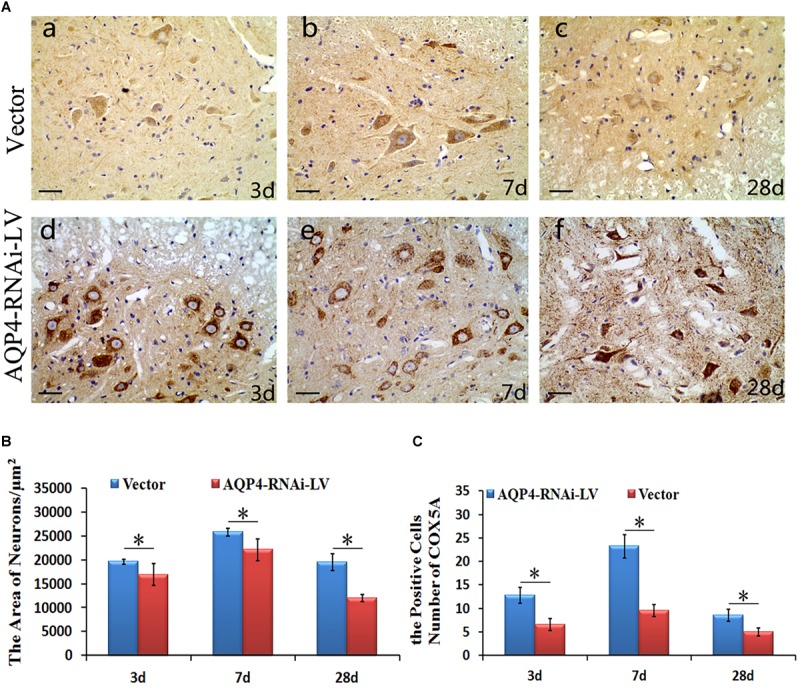
Localization and expression of COX5A and effect of AQP4 inhibition in SCC. **(A)** IHC staining determined the localization of COX5A (brown) in motoneuron mainly following AQP4-RNAi lentivirus transduction. Black arrows mark COX5A. The group of AQP4-RNAi-LV had more obvious COX5A-positive products in motoneurons than the vector groups at 3, 7, and 28 days after SCC. Moreover, at 7 days as well as 28 days, there was a significant loss of COX5A-positive products in both the vector and AQP4-RNAi-LV groups. **(B)** The area of neurons in the AQP4-RNAi group was smaller than the vector groups at 3, 7, and 28 days after SCC. ^∗^denotes *P* < 0.05, compared with vector groups. **(C)** The cell numbers of COX5A expressed in the AQP4-RNAi groups and the vector groups are shown. All of the AQP4-RNAi groups were higher than vector groups. ^∗^denotes *P* < 0.05, compared with Vector groups.

Subsequently, IHC was performed to assess morphological changes of the spinal cord tissue after SCC following AQP4 interference. Compared with the control group, the treatment group partially reversed those changes induced by SCC, including cellular swelling or necrosis and illegible cell borders after SCC (Figure 5A). The areas of neurons in the AQP4-RNAi-LV group were smaller than those in the vector group after 3, 7, and 28 days in SCC (*P* < 0.05, Figure 5B). In addition, all the AQP4-RNAi groups were higher than the vector group in COX5A-positive cells (*P* < 0.05, Figure 5C).

## Discussion

**FIGURE 6:**
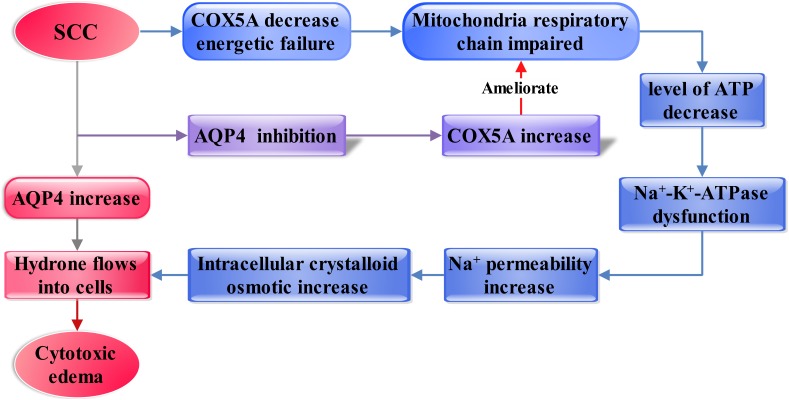
Diagram showing the framework of discussion. The increasing AQP4 influences vascular and cytotoxic edema after SCC (shown as the red and gray arrows). Then, inhibition of AQP4 expression mediated by lentivirus significantly ameliorated cytotoxic edema after SCI. Furthermore, AQP4 is found as a regulator in the cytotoxic edema, probably dependent on regulating the COX5A expressional levels, which may affect membranous Na^+^-K^+^-ATPase by regulation of ATP generation (shown as the blue and purple arrow).

In this study, we established a SCC model in rats that resulted in severe edema in the injured spinal cords. Vasogenic edema at the early stage and cytotoxic edema at the middle stages were observed in injured spinal cords. Limited movement was found in rats after SCC, suggesting a weak repair capacity in the spinal cord following injury. In addition, it was found that AQP4 substantially upregulated after SCC, demonstrated by WB. These suggested that AQP4 might play a crucial role in the formation of cytotoxic edema and limited movement after SCC. In order to elucidate the mechanism of edema that might be associated with AQP4, GeneMANIA bioinformatical analysis was used. It revealed that AQP4 and COX5A were connected by the intermediator Ndufb3, which was further confirmed by immunofluorescence. The BBB test showed that inhibition of AQP4 could accelerate functional recovery in the hindlimbs after SCC. Furthermore, AQP4-RNAi mediated by lentivirus resulted in upregulation of COX5A in crushed spinal cord, as was demonstrated by qRT-PCR and WB. These observations were reasoned as the possibility that the inhibition of AQP4 might influence the formation of cytotoxic edema via upregulating the expression of COX5A, thus promoting motor functional recovery in impaired hindlimbs from SCC. The framework of discussion showed in Figure 6.

### Effect of AQP4 on Vascular and Cytotoxic Edema After SCC

In this study, we observed that AQP4 facilitated the resolution of vasogenic edema by reabsorption of excess fluid in the early stage of SCI. Nevertheless, AQP4 was also found promoting the formation of cytotoxic edema with the developmental stages of SCI. Therefore, AQP4 has dual effects on vasogenic edema and subsequent cytotoxic edema after SCI.

Firstly, we observed that the expression of AQP4 was in VECs, astrocytes, and even in the location of neuronal membranes of the anterior horn in the spinal cord at different times after SCC. In normal spinal cord, the expression of AQP4 was low at a level to maintain the normal physiological function of spinal cord. In the early stage of SCC, AQP4 was mainly expressed in vascular endothelia cells, but rare in astrocytes and neuronal membranes of the anterior horn of spinal cord. Simultaneously, the water content in the spinal cord reached the maximum in 3 days measured by the wet weight method, which suggested that vasogenic edema was the main type of spinal cord edema. Many lines of literature have evidenced that the formation of vasogenic edema was due to the destruction of the tight junctions between endothelial cells and the increase of the permeability of the blood-spinal cord barrier, which suggested that AQP4 was not involved in the formation of vasogenic edema (Manley et al., 2000; Ni et al., 2015; Yang et al., 2015). Other research has also reported that AQP4 was responsible for spinal cord swelling following compression injury with remarkably improved outcome in AQP4 knockout mice (Papadopoulos and Verkman, 2005). Another research demonstrated that AQP4 played a beneficial role in facilitating reabsorption of fluid in vasogenic edema, including some models of TBI, cerebral edema and SCI (Hsu et al., 2015), thus AQP4 reduced the degree of vasogenic edema during this period. In the middle stages of edema, AQP4 was observed around the position of neuronal membranes and in the end-foot of astrocyte after 5–14 days with SCC. Subsequently, the expression of AQP4 decreased in VECs and began to increase around the position of membranes neurons and in glial end-feet of the spinal cord. At this moment, cytotoxic edema was observed clearly in the spinal cord, but it did not continue to expand during the following weeks until 28 days after SCC. Meanwhile, the expression of AQP4 in glial cells and VECs was restored to normal level as it was before. Thus, we reasoned that cytotoxic edema was the major type of spinal cord edema in the later stage of SCC. There was some evidence that change in local tonicity was the key driver of cytotoxic edema following TBI and contributed to the re-localization of AQP4 (Kitchen et al., 2015b). Other research has demonstrated that AQP4 could worsen outcomes of cytotoxic edema in some models, such as TBI and ischemia. Yang et al. (2008) established a model of brain injury and provided evidence for the involvement of AQP4 in enhancing water influx to the cells and promoting acute cytotoxic brain edema. A similar observation was reported whereby the increase of AQP4 expedited the development of cytotoxic edema (Kapoor et al., 2013). In summary, AQP4 participated in the generation and development of cytotoxic edema.

Recent research on the role of AQP4 in spinal cord edema, coming from different animal injury models, showed that the results were contradictory in vasogenic edema and cytotoxic edema. However, in this study we reveal the dual effects of AQP4 in the two types of edema after SCC and assert that AQP4 acts to clear excess fluid in vasogenic edema in the early stages of SCC and aggravates cytotoxic edema by influencing water accumulation in the neurons of the spinal cord in the middle stages of SCC, and it further causes more severe impairments to the spinal cord. A critical role exists for it regarding the ultimate outcomes of pathological conditions in the spinal cord after SCC. In summary, AQP4 has dual roles in spinal cord vasogenic edema and cytotoxic edema in various stages after SCC.

#### The Roles of AQP4 in the Cytotoxic Edema With SCC

In the present study, we have found that inhibition of AQP4 expression mediated by lentivirus could significantly ameliorate cytotoxic edema and the motor function of rats after SCI. Our IHC results show that the cytotoxic edema of neurons is mainly exhibited in the injured part of the spinal cord, with significant damage of nerve cells, such as vague cell borders, cell shrinkage, and necrosis. Cytotoxic edema could also be influenced by hypoxic conditions, and astrocyte edema was the most conspicuous after hypoxic brain injury (Danny et al., 2007; Morken et al., 2014; Mootaz et al., 2017a). The results of qRT-PCR and WB indicate that AQP4 is upregulated significantly after SCC, consistent with other studies that AQP4 is upregulated in edema with various animal models (Nesic et al., 2006; Zador et al., 2009; Verkman et al., 2011; Wolburg et al., 2012). Additionally, this study also shows that AQP4 is abundantly expressed on the membranes of motor neurons, end-feet of astrocytes and capillaries by IHC and immunofluorescence. As the level of AQP4 expression increased, cytotoxic edema develops remarkably in SCC, whereas the extent of edema in injured neurons and other cells is relieved visibly after the inhibition of AQP4. Therefore, our results suggest that AQP4 contributes to the improvement of cytotoxic edema, especially in the neuronal swelling of the contusion of spinal cord. There are some studies given the evidence for the involvement of AQP4 in cytotoxic edema in other injury models. Yang et al. (2015) indicated that the knockdown of the AQP4 gene significantly ameliorated hypoxia-ischemia-mediated cytotoxic edema in the brain neonatal piglet model. Manley and Papadopoulos demonstrated that AQP4 deletion decreased cytotoxic edema in the brain of mice (Manley et al., 2000; Papadopoulos and Verkman, 2005). Li et al. (2013) reported that phosphorylation and expression regulation of AQP4 were involved in the formation of cytotoxic edema in the brain. Further, Saadoun et al. (2008) showed clearly that AQP4-dependent cellular swelling after spinal cord compression injury resulted in a remarkably improved outcome in AQP4-null mice. In some experiments, using mice in the absence of AQP4 showed that AQP4 facilitated edema formation in conditions with cytotoxic edema such as cerebral ischaemia, hyponatraemia, and meningitis (Verkman et al., 2006; Akdemir et al., 2014; Yao et al., 2015). All these reports are consistent with our results. In brief, our studies demonstrate that AQP4 facilitates the formation of cytotoxic edema, which influences the recovery of the motor function in rats after SCI.

### AQP4 Regulates COX5A in Cytotoxic Edema After SCC

From the above discussion we assume that the amelioration of cytotoxic edema is probably related to the upregulation of COX5A through inhibiting the expression of AQP4. Bioinformation analysis shows that AQP4 and COX5A are both related to Ndufb3. Ndufb3 encodes a structural Complex I subunit that is the first enzyme of the mitochondrial respiratory chain (Stroud et al., 2016). Immunofluorescence shows that COX5A is expressed in large motor neurons in the anterior horn of the spinal cord gray matter. Furthermore, our results of qRT-PCR and WB show that the expression of COX5A is decreased significantly after SCC, while AQP4 is increased; in contrast, a dramatic increase of COX5A is seen after AQP4 inhibition. IHC then shows that COX5A predominantly resides in the cytoplasm of motor neurons in the spinal cord and when COX5A expression increased, a significant amelioration of cytotoxic edema emerged. Those suggested that the expression of AQP4 is altered in response to the expression of COX5A along with the change in the extent of cytotoxic edema. Intriguingly, emerging evidence indicates that AQP4 is necessary to mitochondrial functions. Duan et al. (2013) showed that intestinal mitochondria had various degrees of swelling with significant increased expression of AQP4 in TBI. It was also demonstrated that mitochondria-mediated apoptosis was minimized with a prominent decrease in the content of AQP4 following TBI (Lv et al., 2013). Besides, Rama indicated that AQP4 protein increased while glutamine failed to transport into the mitochondria in experimental acute liver failure. Meanwhile, edema was facilitated by the mitochondria (Rama Rao et al., 2010). All these facts strongly suggest the interrelationship between AQP4 and mitochondria.

Additionally, it was well-recognized that cytotoxic edema was associated with the function of Na^+^-K^+^-ATPase, which is necessary for the normal function of mitochondria (Allain et al., 1987; Ito et al., 2003; Hertz et al., 2014). Particularly, Kempski (2001) have proposed that the pathogenicity of cytotoxic edema may be due to the failure of Na^+^ export via Na^+^-K^+^-ATPase because of the energy shortage. Thus, we propose that increase of AQP4 may cause mitochondrial dysfunction associated with Na^+^-K^+^-ATPase, which could result in the increased intracellular crystalloid osmotic pressure and cytotoxic edema ultimately.

In addition, COX5A, a critical enzyme complex of respiratory electron transport chain in mitochondria, may be closely related to the energy metabolism and the functionality of Na^+^-K^+^-ATPase through contributions to the conservation of ATP, the major source of energy for cellular reactions (Allain et al., 1987; Burke et al., 1997; Ito et al., 2003; Castello et al., 2008; Uddin et al., 2008; Rama Rao et al., 2010; Chen et al., 2012; Duan et al., 2013; Hassan and Bilitewski, 2013; Cui et al., 2014; Hertz et al., 2014). Particularly, on this subject, Cui through modulation of the respiratory supercomplexes in the yeast proved that yeast cells with over-expressed cytochrome oxidase (CcO) subunits of COX5A could lead to enhanced respiration (Cui et al., 2014). Beyond that, Pierrel over-expressed COX5A in yeast mitochondria and also detected restored respiration (Pierrel et al., 2008). Briefly, COX5A plays a crucial role in cellular respiration. Additionally, neuron-astrocyte energy metabolism plays a key role in neuronal activity (Magistretti and Allaman, 2015). AQPs may be involved in cell signaling for volume regulation and controlling the subcellular localization of other proteins by forming macromolecular complexes (Kitchen et al., 2015a), AQP9 as a neutral solute channel is implicated in brain energy metabolism (Badaut and Regli, 2004). We hypothesize that the expression of COX5A regulated by AQP4 induces the cytotoxic edema in the spinal cord after SCC and is related to the damage of Na^+^-K^+^-ATPase, further impacting the respiration of mitochondria.

However, there are some limitations on safety and long-term effectiveness of Lentivirus-mediated RNA interference technology in the current research of the gene interference field, which further affected experimental research. Therefore, neuronal culture and CRISPR/Cas9 interference *in vitro* will be used to further validate the correlation of AQP4 and COX5A in the future.

## Conclusion

This study shows that AQP4 plays a dual role in the vasogenic and cytotoxic edema of the spinal cord at different stages of SCI. In the early stage of SCC, increasing AQP4 promotes water reabsorption and improves vasogenic edema. In the middle stages of SCC, AQP4 aggravates the formation and development of cytotoxic edema. The downregulation of AQP4 ameliorates the consequence of cytotoxic edema and ameliorates the motor function after SCC. Furthermore, AQP4 is found as a regulator in cytotoxic edema probably relating to the regulation of COX5A expressional levels, which may affect membranous Na^+^-K^+^-ATPase via regulation of ATP generation. These findings highlight that AQP4-regulated cytotoxic edema in SCC may be associated with energy metabolism via COX5A. Further research in this field may contribute to explore therapeutic approaches to the clinical treatment of secondary injury of SCC, particularly targeting edema in early stage of SCC.

## Ethics Statement

Spinal cord contusion study was conducted in accordance with the National Institutes of Health guidelines for the treatment of animals and all experiments were approved by the Institutional Animal Care and Use of Laboratory Animals of Sichuan University, Chengdu, China.

## Author Contributions

YH and S-nL prepared the manuscript and performed the experiments. X-yZ, G-yC, and Q-jX assisted in conducting many of the experiments. XZ and NL conceived of the idea for the project and contributed to the experimental design. L-xZ and T-hW assisted in the preparation of the manuscript. T-hW provided the AQP4-RNAi lentivirus. XZ provided excellent advice in writing the manuscript.

## Conflict of Interest Statement

The authors declare that the research was conducted in the absence of any commercial or financial relationships that could be construed as a potential conflict of interest.

## Footnotes

1 http://www.genemania.org

## Supplementary Material

FIGURE S1 Laminectomy.

FIGURE S2 Prepare models of SCC.

FIGURE S3 Rats hindlimb motor dysfunction–model preparation succeeded.

FIGURE S4 Spinal cords were inflicted by an Infinite Horizon Device.

TABLE S1 Analysis of BBB score after AQP4-RNAi.

TABLE S2 Analysis of BBB score after SCC.

TABLE S3 Analysis of neurons areas after SCC.

TABLE S4 Analysis of qRT-PCR results.

TABLE S5 Analysis of wet-dry weight after SCC.

TABLE S6 The expression of AQP4 and COX5A in Western blot after SCC.
