# Supplementary figures and images for: The Dual Role of AQP4 in Cytotoxic and Vasogenic Edema Following Spinal Cord Contusion and Its Possible Association With Energy Metabolism via COX5A
Source: Front Neurosci. 2019 Jun 14;13:584. doi: 10.3389/fnins.2019.00584 (PMC6587679; doi:10.3389/fnins.2019.00584)

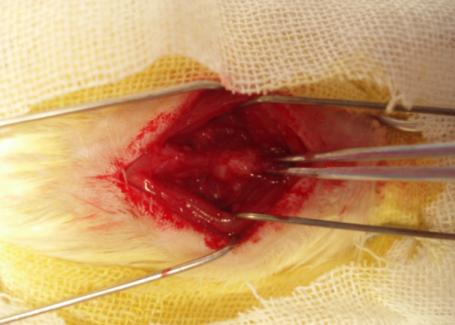

Supplement: FIGURE S1 — Laminectomy. [file Image_1.JPEG]

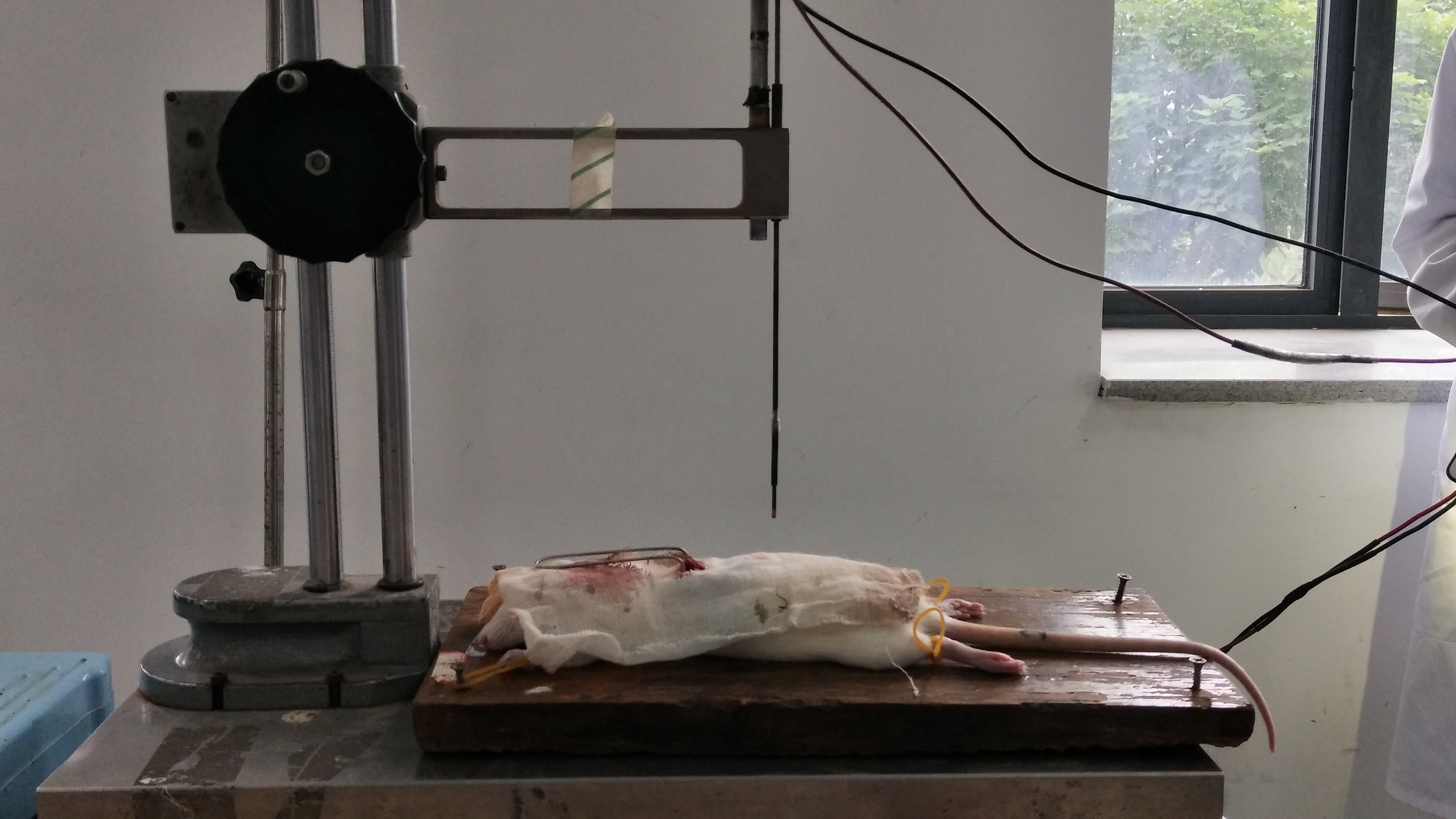

Supplement: FIGURE S2 — Prepare models of SCC. [file Image_2.JPEG]

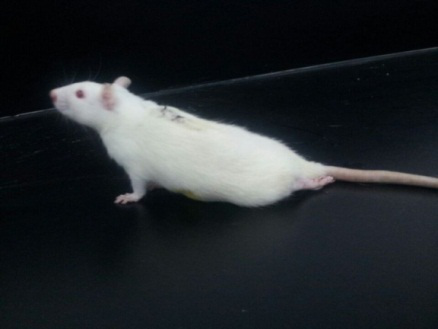

Supplement: FIGURE S3 — Rats hindlimb motor dysfunction–model preparation succeeded. [file Image_3.PNG]

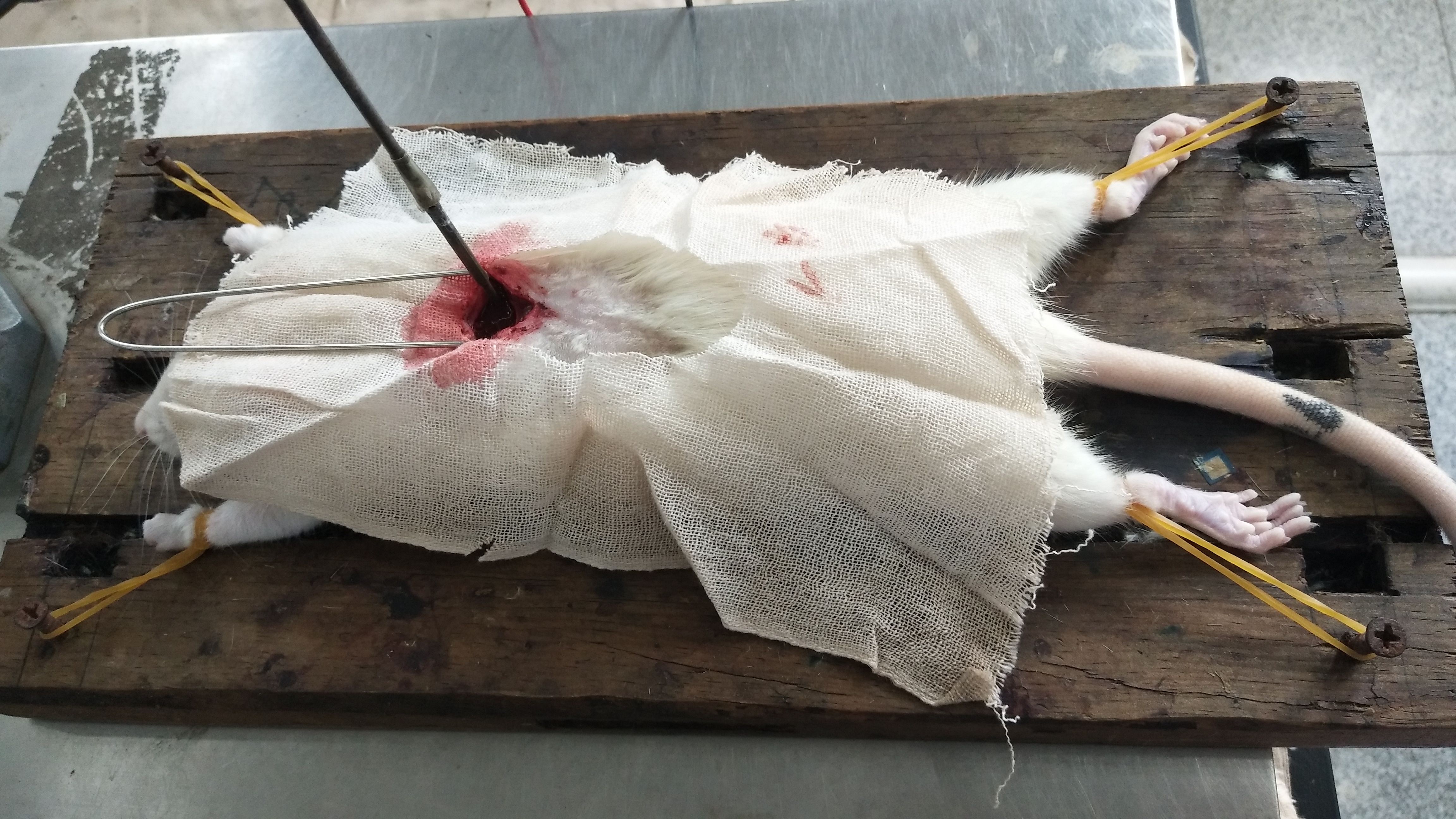

Supplement: FIGURE S4 — Spinal cords were inflicted by an Infinite Horizon Device. [file Image_4.JPEG]
